# Mapping the evidence on public health competency assessment strategies in undergraduate medical education: a scoping review

**DOI:** 10.1186/s12909-026-09976-0

**Published:** 2026-07-21

**Authors:** Erwin Hernando Hernández Rincón, Mariana González Garcés, Jerónimo Cárdenas Montoya, Valeria Concha Fernández, Mario Andrés Torres Torres, Nicolle Valeria Vargas Villafañe, Natalia Castaño Chaves

**Affiliations:** 1 Department of Family Medicine and Public Health, Universidad de La Sabana, Campus Puente del Común, Km 7 Autopista Norte, Chía, Colombia; 2 Primary Care Physician, School of Medicine, Universidad de La Sabana, Chía, Colombia

**Keywords:** Public health, Medical education, Competency assessment, Undergraduate medical education, Social determinants of health

## Abstract

**Background:**

The increasing complexity of health systems and the growing importance of social determinants of health require physicians trained with a population-based perspective. However, assessment of public health competencies in undergraduate medical education remains heterogeneous, with limited conceptual integration and lack of standardised approaches.

**Objective:**

To map and characterise the scientific evidence on strategies used to assess public health competencies in undergraduate medical students.

**Methods:**

A scoping review was conducted following Joanna Briggs Institute methodology and reported according to PRISMA-ScR guidelines. A systematic search was performed in PubMed, Scopus, LILACS and IEEE Xplore for studies published between January 2011 and January 2026. Studies involving undergraduate medical students and addressing assessment of public health competencies were included. Data were extracted using a structured matrix and synthesised narratively.

**Results:**

Thirty studies were included, demonstrating marked heterogeneity across study designs, including randomised controlled trials, observational, qualitative and mixed-methods studies. Assessment strategies were organised into key domains, ranging from knowledge-based evaluations to performance-based approaches in authentic settings. Experiential strategies, such as community-based learning and service learning, enabled integration of social determinants of health into assessment processes. A continuum of evaluative complexity was identified, reflecting a shift from cognitive assessment towards multidimensional, context-based and performance-oriented approaches. Most studies were conducted in high-income countries.

**Conclusions:**

Assessment of public health competencies is evolving towards integrated, contextualised and performance-based models. However, the absence of standardised frameworks and validated instruments limits comparability across studies. Advancing towards longitudinal and programmatic assessment models is essential to strengthen medical training and align it with contemporary population health challenges.

## Introduction

Contemporary health systems face growing challenges arising from population ageing, epidemiological transition and the sustained increase in non-communicable chronic diseases. In parallel, the emergence of global health emergencies has highlighted the need for health professionals capable of addressing health problems from a population-based perspective. The World Health Organization estimates that by 2030 the world will face an approximate deficit of 11 million health workers, a situation that poses significant challenges for health systems and for institutions responsible for training the health workforce [1]. In this context, medical education plays a fundamental role in preparing physicians capable of responding to current and future health challenges.

Concurrently, medical education has experienced considerable expansion worldwide over recent decades. It is estimated that there are currently approximately 2,600 medical schools distributed across more than 200 countries, responsible for training over 1.3 million medical students each year [2, 3]. This growth has generated increasing interest in ensuring the quality of medical training and in ensuring that future doctors develop competencies that enable them to address not only individual disease, but also the social and population determinants of health.

In this regard, public health has become established as an essential component within medical training programmes. Traditionally, medical curricula have been centred on individual clinical care; however, recognition of factors such as health inequalities, social determinants and global epidemiological threats has driven the need to train physicians with a population health perspective. International organisations such as the World Health Organization and the Pan American Health Organization have indicated that health professionals must develop competencies related to epidemiological surveillance, health promotion, disease prevention and understanding of health systems functioning [4, 5].

In the context of undergraduate medical education, public health competencies refer to the knowledge, skills and professional attitudes that enable future physicians to understand and address population health needs. These competencies commonly include epidemiology, disease prevention, health promotion, social determinants of health, health systems understanding, community engagement, health advocacy and population-based decision making. This conceptualisation is consistent with competency frameworks proposed by the Association of Schools and Programs of Public Health and other international medical education organisations [7, 8].

Taking the above into account, numerous medical schools have progressively incorporated public health content within their undergraduate programmes. These strategies include courses in epidemiology, preventive medicine, community health and global health, as well as community-based learning experiences [12, 17, 19]. Nevertheless, various studies have shown that the level of knowledge and competencies in public health amongst medical students continues to be variable. Recent research has reported that a significant proportion of students have limited knowledge on topics related to global health and social determinants of health, which reveals gaps in population health training within medical curricula [6].

However, the assessment of public health competencies within undergraduate medical education represents a pedagogical and methodological challenge. Unlike other clinical areas, many public health competencies involve complex skills related to the analysis of epidemiological information, understanding of social determinants, planning of community interventions and interdisciplinary work [7]. Consequently, traditional assessment methods based exclusively on written examinations may prove insufficient to adequately evaluate the development of these competencies. In response to this problem, various educational institutions have begun to implement broader assessment strategies, including academic portfolios, community projects, outbreak investigations, problem-based learning and structured performance assessments [25, 37, 44].

However, despite growing interest in public health training within medical education, the available literature shows considerable heterogeneity in the strategies used to assess these competencies. Different conceptual frameworks have proposed competency domains that include areas such as population data analysis, understanding of health systems, health promotion and community participation [8]. Nevertheless, there is still a lack of international consensus regarding the most appropriate methods for assessing these competencies during medical training. Furthermore, many of the reported educational experiences are described in isolation, which makes it difficult to obtain a comprehensive view of the strategies used in different educational contexts.

Given this heterogeneity, it is necessary to synthesise the available evidence on public health competency assessment strategies in undergraduate medical education. Scoping reviews constitute an appropriate methodology for mapping complex or emerging research fields, as they allow identification of the types of available evidence, methodological approaches used and existing gaps in the scientific literature [9]. In this context, a scoping review can provide a comprehensive overview of the strategies employed to assess public health competencies within medical training programmes.

Therefore, this study aims to map and characterise the strategies used to assess public health competencies in undergraduate medical students, in order to identify the assessment methods implemented, the educational contexts in which they are applied, and the competency dimensions evaluated.

## Methods

### Study design

A scoping review was conducted with the aim of mapping and characterising the available scientific evidence on the strategies used to assess public health competencies in undergraduate medical education. This design was selected for its suitability in synthesising methodologically heterogeneous evidence and identifying patterns, approaches and knowledge gaps in a complex field that integrates medical education with population health.

The review was conducted in accordance with the Joanna Briggs Institute methodological guidance and reported following PRISMA-ScR recommendations. The review protocol was prospectively registered in the Open Science Framework (OSF) to enhance transparency and methodological rigour, and is available at: https://osf.io/gqb29.

As the study was designed as a scoping review, no formal assessment of risk of bias or methodological quality of the included studies was performed.

### Research question and PCC framework

The research question was structured using the Population, Concept and Context framework.

The population included students enrolled in undergraduate medicine programmes leading to qualification as physicians (medical degree programmes). Studies involving nursing, allied health, public health, or other health professions students were excluded unless results for medical students were reported separately.

The concept comprised strategies used for the assessment of public health competencies, including methods for assessing knowledge, skills, performance and multidimensional competency-based assessment approaches.

The context included medical education programmes in which public health training and assessment is implemented at undergraduate level.

The research question was defined as follows: What scientific evidence exists on the strategies used to assess public health competencies in medical students during undergraduate medical education?

### Eligibility criteria

Explicit inclusion and exclusion criteria were established prior to the screening process, in accordance with the Population, Concept and Context framework.

Studies were included if they evaluated undergraduate medical students and described or analysed strategies used for the assessment of public health competencies. Only original research with empirical data, published in English or Spanish between January 2011 and January 2026, was considered.

Studies were excluded if they focused exclusively on postgraduate education or on other health professions without specific analysis of medical students, as well as those that addressed public health teaching without including an assessment component. Narrative reviews, systematic reviews, protocols, editorials, letters to the editor and case reports were also excluded.

### Search strategy and information sources

A comprehensive bibliographic search was conducted in PubMed, Scopus, LILACS and IEEE Xplore using predefined keywords and controlled vocabulary (where applicable). The search strategy incorporated keywords representing the main domains of public health competencies, including epidemiology, population health, community health, global health, health promotion, social determinants of health, health advocacy, and health systems, together with competency assessment terminology. The inclusion of IEEE Xplore aimed to capture studies related to technology-mediated educational strategies, including digital learning, e-learning and innovative assessment approaches in medical education. Studies published between January 2011 and January 2026 in English or Spanish were included.

The lower temporal limit was established in order to capture contemporary development of competency-based education and the evolution of assessment strategies in public health within medical training. The upper limit corresponded to the date of the last search.

The complete search strategies used for each database are presented in Table 1. Additionally, backward citation tracking was performed by manually examining the reference lists of the included studies to identify potentially eligible publications. Forward citation tracking was not undertaken. All records were managed using the Rayyan platform.

**Table 1:** Search strategy

Database	Preliminary Search Strategy
PubMed	((“medical student*” OR “undergraduate medical education”) AND (“public health”) AND (competenc*) AND (“competency assessment” OR “competence assessment” OR assess* OR evaluat*))
IEEE XPLORE	(“medical education” OR “medical student” OR “medical students” OR “medical undergraduate” OR “health professions education”) AND (“public health” OR “community health” OR “population health” OR epidemiology) AND (competenc* OR skill* OR learning OR training OR curriculum) AND (assessment OR evaluation OR measur* OR examin* OR testing)
Scopus	TITLE-ABS-KEY ( “public health” OR “population health” OR “community health” ) AND TITLE-ABS-KEY ( competenc* ) AND TITLE-ABS-KEY ( assessment OR evaluation OR examination ) AND TITLE-ABS-KEY ( “medical student*” OR “undergraduate medical education” ) AND PUBYEAR > 2010 AND PUBYEAR < 2027
Lilacs	(“public health” OR “community health” OR “population health” OR “salud pública”) AND (competenc* OR “professional competence” OR competencia*) AND (assessment OR evaluation OR “competency assessment” OR “competency evaluation” OR evaluacion) AND (“medical student*” OR “undergraduate medical education” OR “medical undergraduate*” OR “educación médica” OR estudiantes OR pregrado)

### Study selection

Following identification of records and removal of duplicates, studies were independently evaluated during title and abstract screening by two reviewers (M.G.G. and J.C.M.), both physicians with experience in evidence synthesis and medical education research. Studies meeting the preliminary eligibility criteria underwent independent full-text assessment by the same reviewers. Discrepancies between reviewers were resolved through discussion and consensus. When consensus could not be reached, a senior reviewer (E.H.H.R.), with expertise in public health and medical education research, was consulted to reach a final decision.

**Fig. 1:**
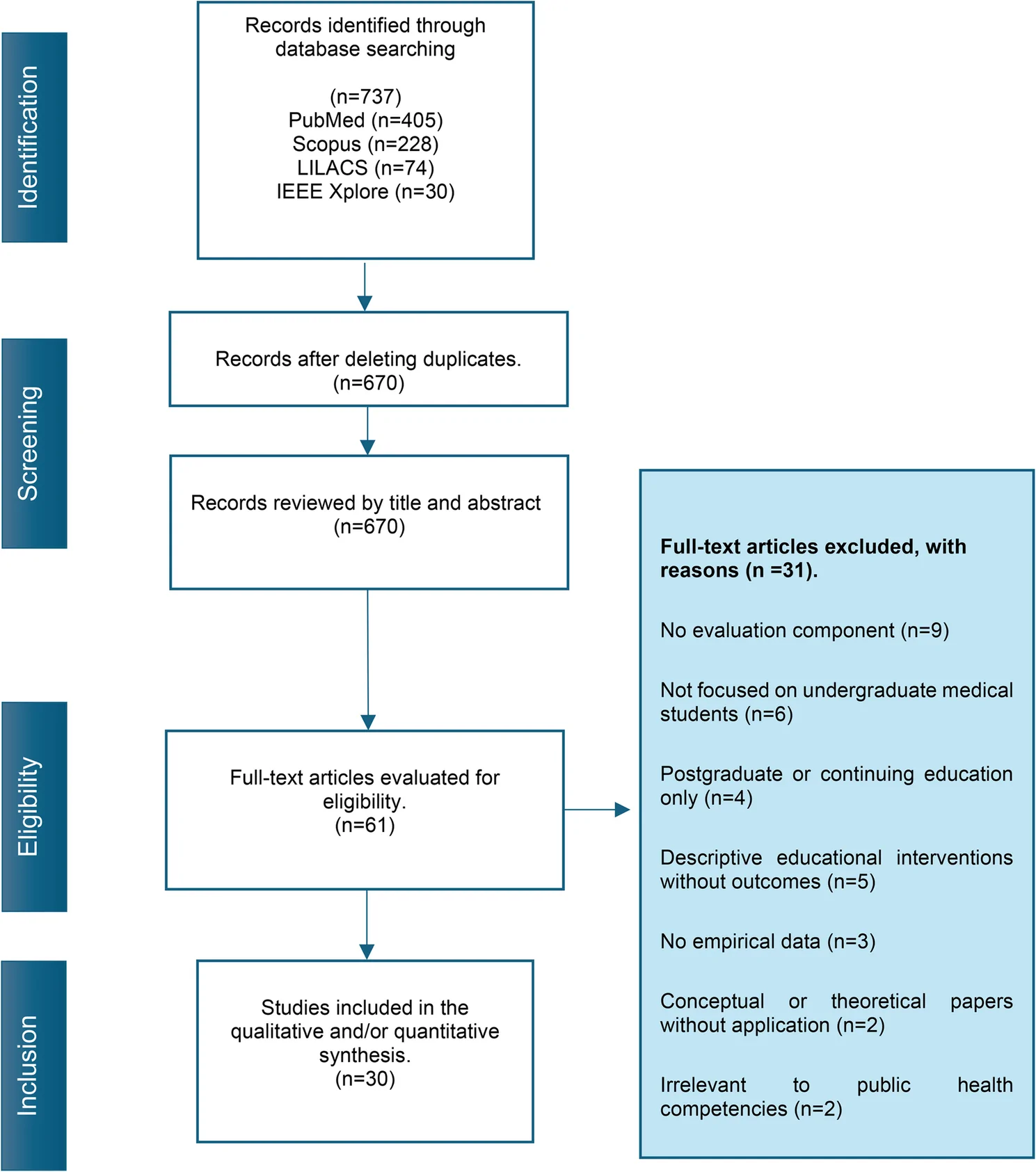
Prisma-ScR

The complete process of identification, screening, eligibility and inclusion is summarised in the PRISMA-ScR flow diagram (Fig. 1).

### Data extraction, analysis and synthesis

Data extraction was performed using a structured matrix designed in Microsoft Excel. Variables recorded included general characteristics of studies, educational context, type of public health competency assessed, assessment strategies employed, instruments used and reported outcomes.

For synthesis purposes, central variables corresponded to the type of assessment strategy, competency dimensions addressed and the relationship between evaluative methods and competency-based education approaches. Other variables were used for descriptive characterisation of included studies.

A narrative synthesis was conducted, grouping studies according to the type of assessment strategy and their contribution to the development of public health competency assessment processes in undergraduate medical education.

## Results

### Study selection

The search strategy identified a total of 737 records through electronic databases, distributed across PubMed (*n* = 405), Scopus (*n* = 228), LILACS (*n* = 74) and IEEE Xplore (*n* = 30). Following removal of duplicates using the Rayyan platform, 670 unique records were retained for title and abstract screening.

Subsequently, 61 articles were evaluated as full text, of which 31 were excluded for not meeting the previously defined eligibility criteria. Finally, 30 studies were included in the qualitative synthesis. The complete flow of the selection process is presented in Fig. 1.

The main reasons for exclusion in the full-text phase included the absence of an explicit component of public health competency assessment, focus on educational interventions without outcome measurement, inclusion of populations other than medical students, exclusive focus on postgraduate education, lack of original empirical data, as well as the exclusively conceptual character of some studies without direct evaluative application.

### General characteristics and methodological configuration of the evidence

The 30 included studies demonstrate notable methodological heterogeneity, encompassing randomised controlled trials [10], observational studies [11], curricular evaluations [12, 16, 22, 23], mixed-methods studies [17, 23], programme evaluations [15, 20, 32, 33], qualitative studies [31], institutional surveys [25] and conceptual or consensus developments [38, 44].

Table 2 summarises the characteristics of the included studies. Publications ranged from 2014 to 2025 and were conducted across ten countries, with the United States contributing the largest number of studies. The evidence encompassed a broad range of methodological designs, including randomized controlled trials, observational studies, curricular evaluations, mixed-methods studies, qualitative research, programme evaluations, surveys, framework development studies and expert consensus processes. Public health competency domains most frequently addressed included population health, global health, social determinants of health, community health, health advocacy, health literacy and public health practice.

**Table 2:** Characteristics of the included studies

Study	Year	Country	Study design	Training stage	Public health competency domain	Assessment strategy
Zhang et al. [10]	2023	China	Randomized controlled trial	Undergraduate	Epidemiology	Community-based assessment
Baker et al. [11]	2020	United States	Observational study	Undergraduate	Community health	Community health assessment project
Prunuske & Remington [12]	2017	United States	Curricular evaluation	Undergraduate	Population health	Community health assessment curriculum
Essa-Hadad et al. [13]	2015	United Kingdom	Educational evaluation	Undergraduate	Population health	Service learning
Belkowitz et al. [14]	2014	United States	Comparative study	Undergraduate	Health advocacy	Curricular module
McGrew et al. [15]	2015	United States	Programme evaluation	Clinical	Health policy and advocacy	Clerkship assessment
Gonzalez et al. [16]	2015	United States	Curricular evaluation	Undergraduate	Health disparities	Elective programme evaluation
Gruner et al. [17]	2015	Canada	Mixed-methods study	Undergraduate	Global health	E-learning programme
Ambrose et al. [18]	2017	Australia	Pilot study	Undergraduate	Global health	Online collaborative learning
Biswas et al. [19]	2020	Canada	Educational evaluation	Undergraduate	Global health	Patient-based learning
Altillo et al. [20]	2021	United States	Educational evaluation	Clinical	Global health	Elective programme evaluation
Kim et al. [21]	2024	South Korea	Educational evaluation	Undergraduate	Global health	Competency-based programme
Roper et al. [22]	2024	United States	Curricular evaluation	Preclinical	Health equity	Equity-focused curriculum
Finnie et al. [23]	2021	United States	Mixed-methods study	Preclinical	Social determinants of health	Social medicine curriculum
Kneer et al. [24]	2023	United States	Educational evaluation	Preclinical	Social determinants of health	Educational intervention
Campbell et al. [25]	2022	United States	National survey	Mixed/Institutional	Social determinants of health	Assessment survey
Bickerton et al. [26]	2020	United States	Programme evaluation	Clinical	Social determinants of health	Service learning
Wong et al. [27]	2022	Canada	Educational evaluation	Undergraduate	Community health	Service learning
Sheehan et al. [28]	2023	United States	Instrument evaluation	Undergraduate	Social determinants of health	SSDH-CAT validated tool
Dash et al. [29]	2022	Canada	Curricular evaluation	Undergraduate	Social accountability	Problem-based learning
Rahma et al. [30]	2023	United Arab Emirates	Educational evaluation	Clinical	Public health	Clerkship assessment
Ghazali & Soleman [31]	2023	Malaysia	Qualitative study	Clinical	Public health	Clinical rotation evaluation
Demmer et al. [32]	2024	Germany	Programme evaluation	Clinical	Public health	Elective evaluation
Haruta et al. [33]	2025	Japan	Programme evaluation	Clinical	Community health	Fieldwork assessment
Hildenbrand et al. [34]	2020	United States	Educational evaluation	Undergraduate	Health literacy	Communication training assessment
Marion et al. [35]	2018	United States	Educational evaluation	Clinical	Health literacy	OSCE and standardized patient assessment
Coleman et al. [36]	2016	United States	Longitudinal study	Undergraduate	Health literacy	Longitudinal follow-up assessment
Jain et al. [37]	2025	United States	Course evaluation	Undergraduate	Public health	Written examinations
Johnson et al. [38]	2020	United States	Framework development	Not applicable*	Population health	Evaluation framework
Mangold et al. [44]	2023	United States	Expert consensus	Not applicable*	Social determinants of health	Delphi consensus recommendations

*Abbreviations*: *OSCE* Objective Structured Clinical Examination, *SSDH-CAT* Social and Structural Determinants of Health Curriculum Assessment Tool

*Not applicable: Framework development and expert consensus studies were not conducted within a specific stage of undergraduate medical training

Despite this diversity, the evidence shows conceptual convergence towards the assessment of public health competencies as a multidimensional process that integrates knowledge, skills and attitudes within specific educational and social contexts. In this regard, studies are organised not only according to isolated evaluative instruments, but around pedagogical structures that articulate the type of competency, learning context and evaluative purpose.

Table 3 synthesises this conceptual architecture, organising studies according to evaluative domains, predominant strategies, competency level and educational contribution. This classification allows evidence of the coexistence of multiple evaluative approaches, from traditional knowledge-centred models to complex strategies based on performance in real scenarios [10–38].

**Table 3:** Structural typology of public health competency assessment strategies in undergraduate medical education

Evaluative Domain	Central Evaluative Logic	Representative Strategies	Level of Competency Evaluated	Predominant Context	Assessment Instruments	Principal Educational Contribution	References
Experiential evaluation in real-world contexts	Performance-based assessment in authentic scenarios	Community projects, service learning, fieldwork, patient-based learning	Skills, attitudes, applied competencies	Community	Project assessment, practical evaluation, realistic assessment	Integrates social determinants and situated learning	[10–13, 19, 26, 27, 33]
Evaluation in clinical settings	Assessment of performance in healthcare practice	Clerkships, public health rotations	Clinical skills, decision-making, applied competencies	Clinical	Surveys, structured assessments, interviews	Facilitates transition to professional practice	[15, 30, 31]
Structured curricular evaluation	Systematic measurement within the formal curriculum	Courses, CBME programmes, modules, electives	Knowledge, skills, attitudes	Course / curriculum	Examinations, validated instruments, structured assessment	Enables standardisation and comparability	[10, 12, 16, 21, 37]
Assessment of competencies in equity and social determinants	Assessment of sociostructural competencies	SDOH curricula, equity, social medicine	Knowledge, attitudes	Preclinical / curriculum	Surveys, programme evaluation, validated tools	Incorporates social justice approach	[22–25, 28, 29]
Assessment through structured simulation	Objective assessment of specific skills	OSCE, clinical simulation, communication training	Skills	Simulation	SP assessment, rubrics	High objectivity and reproducibility	[34, 35]
Technology-mediated assessment	Assessment in digital and collaborative environments	E-learning, virtual learning, online intercultural activities	Knowledge, skills	Virtual	Questionnaires, surveys	Scalability and global learning	[17, 18]
Longitudinal learning assessment	Temporal monitoring of competency development	Longitudinal studies, curricular follow-up	Knowledge	Curriculum	Longitudinal surveys	Evidence of sustained impact	[36]
Institutional and systems evaluation	Analysis of evaluative strategies at the macro level	Institutional surveys, programme evaluation	Assessment methods	Institutional	Surveys, comparative analysis	Enables educational benchmarking	[25]
Conceptual and normative evaluation	Development of theoretical frameworks and standards	Frameworks, consensus, Delphi	Not applicable	Medical education	Conceptual models, Delphi	Defines international standards and guidelines	[38, 44]

### Geographical distribution of the evidence

Scientific production was concentrated predominantly in high-income countries, with the United States contributing the largest number of studies (*n* = 15) [11, 12, 14–16, 20, 22–26, 28, 34–38], followed by Canada (*n* = 4) [17, 19, 27, 29]. The remaining studies were conducted in the United Kingdom (*n* = 1) [13], Australia (*n* = 1) [18], South Korea (*n* = 1) [21], Germany (*n* = 1) [32], Japan (*n* = 1) [33], Malaysia (*n* = 1) [31], the United Arab Emirates (*n* = 1) [30], and China (*n* = 1) [10].

**Fig. 2:**
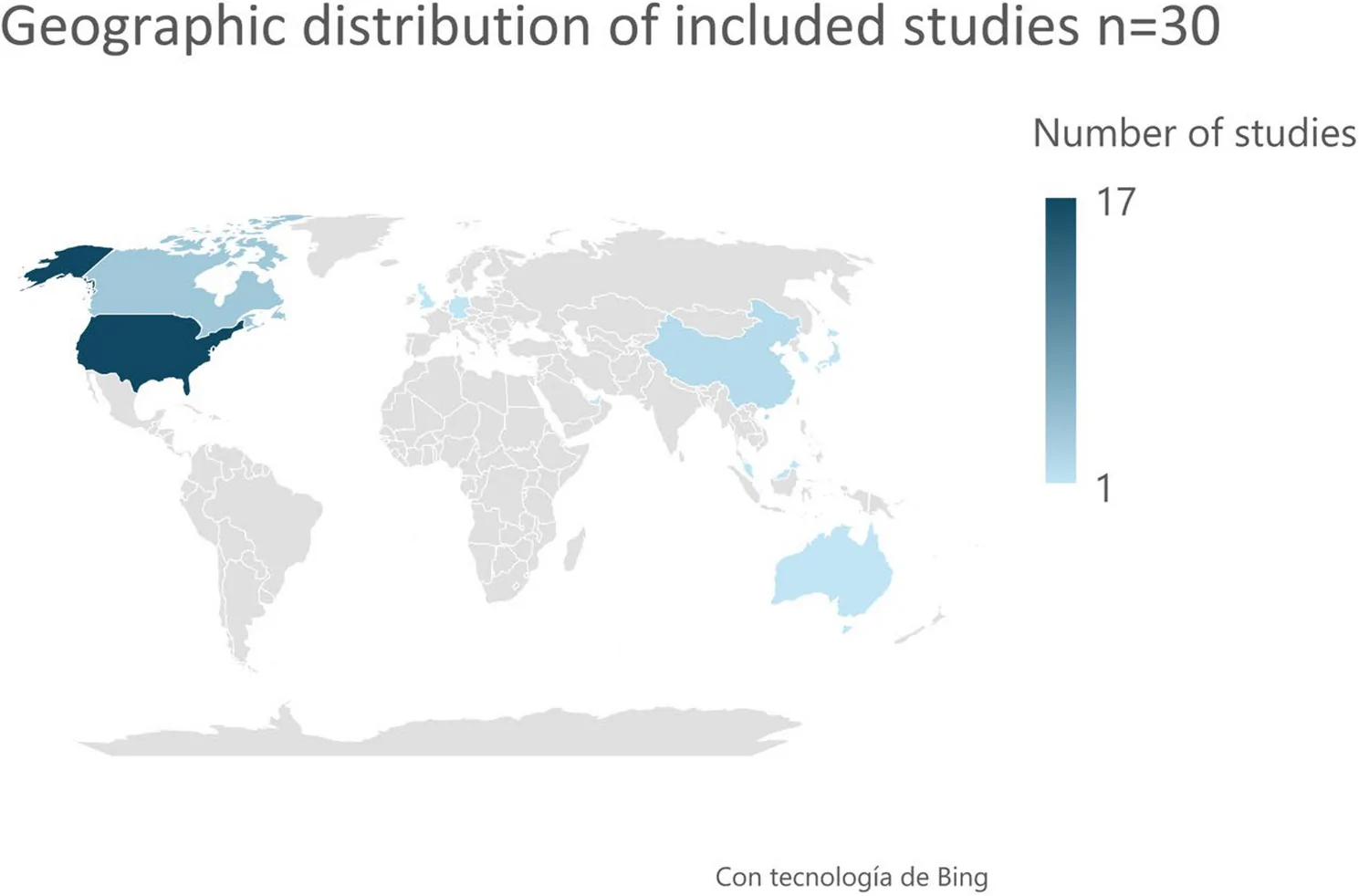
Geographic distribution of included studies

The geographical distribution of studies is presented in Fig. 2 through a heat map, which shows a clear concentration in North America and Europe, with lesser representation in Asia and absence of studies from Latin America and Africa.

This pattern suggests that evidence generation in public health competency assessment is conditioned by the availability of institutional resources, competency-based curricular development and research capacity in medical education, which raises important implications in terms of equity and transferability of findings [10–38].

### Structural typology of assessment strategies

The analysis allowed identification of a structural typology of assessment strategies that transcends classification by individual instruments, organising itself into evaluative domains that reflect different levels of integration between competency, context and method (Table 3).

Strategies based on experience in real contexts constituted one of the most representative domains. These included community projects, service learning, patient-based learning and fieldwork activities, in which assessment is carried out in authentic settings that allow integration of clinical, social and population dimensions [11–13, 19, 26, 27, 33]. In these studies, assessment is oriented not only towards academic performance, but also towards community impact and development of competencies related to social determinants of health [11, 26, 27].

Structured curricular assessment strategies grouped interventions implemented within courses, modules and formal programmes, including competency-based models. These strategies employed standardised instruments such as examinations, structured assessments and validated instruments, which allowed systematic and reproducible measurement of educational outcomes [10, 12, 16, 21, 37].

In clinical settings, particularly through clerkships and public health rotations, assessment focused on performance in real care scenarios, facilitating integration between theoretical knowledge and professional practice [15, 30, 31]. These strategies represent a point of convergence between medical education and health care.

An emerging and highly relevant domain corresponds to assessment of competencies in social determinants of health, equity and social accountability. Studies in this area incorporate socio-structural dimensions within assessment, evidencing a reorientation towards more comprehensive training models [22–25, 28, 29].

Additionally, assessment through structured simulation, including OSCEs and use of standardised patients, allowed objective measurement of specific skills, particularly in health communication and health promotion [34, 35].

Assessment mediated by digital technologies, such as e-learning and virtual collaborative learning, facilitated expansion of educational reach and incorporation of intercultural competencies [17, 18].

Finally, complementary approaches were identified such as longitudinal assessment of learning [36], institutional assessment of educational strategies [25], and development of conceptual frameworks and expert consensus oriented towards standardisation of assessment [38], which reflects a process of progressive maturation of the field.

### Evaluative depth and complexity gradient

Analysis of the identified strategies allowed establishment of a continuum of evaluative complexity, from approaches centred on knowledge to integrated models of assessment in real contexts (Table 4).

**Table 4:** Evaluation depth and educational impact of public health competency assessment strategies

Level of Assessment	Nature of the Measurement	Result Evaluated	Evaluative Complexity	Educational Value Generated	Main Limitations	References
Level 1: Cognitive assessment	Measurement of theoretical knowledge	Knowledge	Low	Enables broad coverage and standardisation	Limited practical applicability	[10, 17, 21, 37]
Level 2: Skills assessment	Performance assessment in specific tasks	Skills	Moderate	Objectifies technical and communicative competencies	May be artificial if not contextualised	[34, 35]
Level 3: Attitudinal assessment	Assessment of perceptions and values	Attitudes	Moderate	Integrates ethical and social dimensions	Subjectivity	[13, 22, 29]
Level 4: Real performance-based assessment	Assessment in authentic scenarios	Skills, impact	High	Meaningful and contextualised learning	Difficult standardisation	[11, 19, 26, 27, 33]
Level 5: Applied clinical assessment	Assessment in healthcare practice	Integrated competencies	High	Links training with medical practice	Contextual variability	[15, 30, 31]
Level 6: Programmatic assessment	Assessment of complete educational interventions	Educational outcomes	Moderate-High	Enables curricular redesign	Methodological heterogeneity	[12, 23, 24]
Level 7: Population/institutional assessment	Assessment at educational systems level	Assessment methods	Moderate	Generates comparative evidence	Does not measure individual learning	[25]
Level 8: Standardised assessment using instruments	Use of validated tools	Competency mapping	High	Improves validity and reproducibility	Contextual limitation	[28]
Level 9: Longitudinal assessment	Measurement over time	Retention and evolution	High	Evaluates sustainability of learning	Costly and complex	[36]
Level 10: Normative assessment and consensus	Definition of standards	Recommendations	High (theoretical)	Guides the educational field	Does not directly assess students	[38, 44]

Complexity levels represent an inductive conceptual classification developed from the characteristics of the included studies and should not be interpreted as a validated assessment framework

At initial levels, strategies oriented towards cognitive assessment predominate, based on examinations and questionnaires, particularly in areas such as epidemiology and global health [10, 17, 21, 37]. These approaches allow measurement of knowledge acquisition, but present limitations in capturing practical application of competencies.

At intermediate levels are strategies oriented towards assessment of skills and attitudes, including clinical simulation, OSCEs, perception surveys and formative assessments [13, 22, 29, 34, 35]. These allow incorporation of behavioural and communicative dimensions, although with a certain degree of artificiality compared to real contexts.

At the most advanced levels, strategies based on performance in authentic scenarios predominate, such as community projects, service learning and clinical rotations [11, 19, 26, 27, 30, 33]. These strategies allow integrated assessment of technical, social and contextual competencies, constituting the most relevant approaches for public health training.

Complementarily, strategies of programmatic and institutional assessment are identified [24, 25], as well as use of validated instruments for competency mapping [28], which reflects progressive methodological sophistication.

At the highest level of abstraction, studies based on consensus and framework development [38] seek to establish global standards, although their implementation remains limited.

### Integrated synthesis of dominant patterns

Integration of findings, supported by the conceptual structure of Table 3 and the complexity gradient described in Table 4, allows identification of three dominant patterns in the assessment of public health competencies in undergraduate medical education.

Firstly, a progressive transition is evident from evaluative models centred on knowledge towards strategies based on performance and experiential learning, particularly in community and clinical contexts [11–13, 19, 26, 27, 30, 33].

Secondly, competencies related to social determinants of health, equity and social accountability emerge as central axes of assessment, reflecting an expansion of the educational approach towards population and structural dimensions of health [22–25, 28, 29].

Thirdly, increasing methodological diversification is observed, with integration of quantitative, qualitative, mixed and conceptual approaches [17, 23, 25, 31, 38]. Nevertheless, this diversity is accompanied by marked heterogeneity in assessment methods, which limits comparability between studies and highlights the need to advance towards more standardised evaluative frameworks.

Overall, the results, synthesised in Tables 3 and 4; Fig. 2, position public health competency assessment as a field under consolidation, characterised by pedagogical innovation, growing methodological complexity and progressive integration between clinical training and population health, although still with significant challenges in terms of standardisation, validation and transferability of evaluative strategies [10–38].

## Discussion

### Conceptual evolution of public health competency assessment in medical education

The evidence synthesised in this review suggests that the assessment of public health competencies in undergraduate medical education has evolved from knowledge-centred approaches towards more integrated models that articulate performance, context and educational meaning.

The included studies demonstrate that these competencies cannot be adequately captured through isolated traditional instruments, but rather require evaluative frameworks capable of integrating clinical, social and population dimensions simultaneously [10, 12, 16, 22, 38]. This transition is consistent with previous reviews that have documented the need to incorporate broader approaches for teaching and assessment of social determinants of health and population competencies in medical education [39, 40].

In this regard, the conceptual architecture presented in Table 3 shows that assessment is not structured solely according to specific tools, but around pedagogical logics that determine what is assessed, how it is assessed and in what context learning occurs. Particularly, strategies based on experiences in real contexts, such as community projects and service learning, emerge as approaches that allow operationalisation of complex competencies related to social determinants, equity and social accountability [11–13, 19, 26, 27, 33].

### Complexity gradient in evaluative strategies

Structural analysis of the evidence, synthesised in Table 4, allows conceptualisation of public health competency assessment as a continuum of progressive complexity. The proposed complexity gradient was developed inductively from the characteristics of the included studies rather than derived from an existing validated framework. Complexity levels were assigned according to the degree of contextual authenticity, integration of multiple competency domains, educational scope, and proximity of assessment to real-world professional performance. Therefore, the gradient should be interpreted as a conceptual synthesis intended to facilitate understanding of the progressive sophistication of assessment strategies rather than as a formal psychometric classification.

At initial levels, approaches centred on cognitive measurement predominate, especially in areas such as epidemiology and global health [10, 17, 21, 37]. These models, although reproducible, present limitations in capturing contextual application of competencies.

At intermediate levels, strategies based on simulation, OSCEs and attitude assessment incorporate behavioural and communicative dimensions [13, 22, 29, 34, 35]. However, these approaches do not always succeed in reflecting the complexity of real environments.

At the most advanced levels, strategies based on performance in authentic scenarios predominate [11, 19, 26, 27, 30, 33], which align with contemporary approaches to competency-based education. This progression is coherent with recent frameworks that propose programmatic assessment as an integrative model for medical training, where multiple methods combine to capture longitudinal student development [44].

### Incorporation of social determinants of health as evaluative axis

One of the most consistent findings of this review is the growing incorporation of social determinants of health as a central component in competency assessment [22–25, 28, 29].

This result aligns with literature that emphasises the need to integrate these determinants systematically within medical curricula [39, 43]. In particular, expert consensus has highlighted that assessment of these competencies requires specific tools that allow measurement not only of knowledge, but also of applied skills and professional attitudes in complex contexts [43, 44].

Likewise, recent reviews have demonstrated that educational interventions oriented towards cultural competencies and social determinants require multimodal evaluative approaches to capture their real impact [40, 41].

However, the findings of this review show that operationalisation of these competencies continues to be heterogeneous, with variability in instruments used and evaluative approaches, which limits comparability between studies.

### Methodological variability and need for standardisation

Despite the conceptual and methodological richness identified, the evidence presents considerable heterogeneity in terms of designs, instruments and assessed outcomes [17, 23, 25, 31, 38].

This variability has been widely documented in previous reviews, which indicate the absence of global standards for assessment of public health competencies and social determinants [39, 40, 41]. In particular, the lack of standardised evaluative frameworks and validated instruments limits the possibility of comparing results between institutions and educational contexts.

Studies oriented towards consensus and framework development [43, 44] represent important advances towards standardisation, by proposing conceptual structures and recommendations for assessment of these competencies. Nevertheless, their implementation remains limited and uniform adoption at global level is not observed.

### Implications for curricular design and medical training

The findings of this review have relevant implications for curricular design in medical education. In particular, they suggest the need to advance towards programmatic assessment models that integrate multiple strategies, contexts and moments of the training process.

Recent literature emphasises that assessment of public health competencies must be longitudinal, multimodal and aligned with curricular objectives [44]. In this regard, integration of performance-based strategies, contextualised assessment and multiple information sources could improve the validity and utility of assessment [24, 25, 28].

Additionally, training in competencies related to social determinants, equity and social accountability has been identified as an essential component for preparing future physicians to face contemporary challenges in global health [39, 42].

### Geographical distribution and evidence generation gaps

The geographical distribution of studies, represented in Fig. 2, demonstrates a predominant concentration in high-income countries [10–38].

The absence of studies from Latin America and Africa is consistent with previous analyses that have identified inequities in knowledge production within medical education [39, 40]. This gap may reflect disparities in research funding, institutional capacity for educational research, publication bias favouring high-income settings, and differences in curricular priorities across regions. It may also be influenced by the underrepresentation of locally conducted studies in indexed international databases. Consequently, the current evidence base may not adequately capture assessment strategies that are responsive to the educational contexts and public health priorities of low- and middle-income countries. This geographical imbalance limits the global applicability and transferability of existing evidence and highlights the need to strengthen context-specific research on public health competency assessment across underrepresented regions.

### Conceptual integration of findings

Synthesis of results, supported by the structure presented in Table 3, the complexity gradient described in Table 4 and the geographical distribution shown in Fig. 2, allows conceptualisation of public health competency assessment as a multidimensional system.

This system integrates competency domains, evaluative strategies and levels of curricular integration, in accordance with recent models that propose a systemic approach to assessment in medical education [44].

### Limitations of primary evidence

The available evidence presents relevant structural limitations that must be considered in interpretation of findings. Firstly, marked heterogeneity is observed in study designs, including controlled trials, observational studies, curricular evaluations, qualitative studies and conceptual developments, which makes comparability between investigations difficult and limits the possibility of establishing robust inferences about the relative effectiveness of different evaluative strategies [17, 23, 25, 31, 38].

Secondly, there is significant variability in the definition and operationalisation of public health competencies, particularly in domains such as social determinants of health, equity and social accountability. This lack of conceptual standardisation translates into use of heterogeneous instruments, many of which lack formal validation, which introduces uncertainty in measurement of educational outcomes and limits reproducibility of results [39–41, 43, 44].

Additionally, a considerable proportion of studies are based on indirect measures, such as student perceptions or self-assessments, rather than objective performance indicators, which increases the risk of social desirability bias and limits internal validity of findings. Likewise, the absence of longitudinal assessments in most studies prevents determination of sustainability of learning and its transfer to real clinical practice.

Finally, concentration of evidence in high-income countries introduces a geographical bias that restricts generalisation of results to contexts with different health system structures, educational resources and sociocultural realities, which is particularly relevant in the field of public health, where context plays a determining role in professional practice.

### Limitations of the present review

As a scoping review, this study had a primarily descriptive and evidence-mapping objective; therefore, no formal assessment of risk of bias or methodological quality of the included studies was performed. Consequently, interpretation of findings depends on the quality and rigour of the available primary evidence.

Likewise, restriction to specific databases, to studies published in English and Spanish, and to the period between 2011 and 2026 may have excluded relevant studies published outside this temporal interval, in other languages, or in non-indexed sources. Although PubMed, Scopus, LILACS and IEEE Xplore were selected to capture literature from medical, public health, educational and technology-oriented domains, additional databases such as ERIC, PsycINFO or Web of Science may have identified further relevant studies. Furthermore, forward citation tracking was not performed, which may have resulted in omission of additional relevant studies that cited the included publications.

Moreover, heterogeneity in study designs, pedagogical approaches and assessment methods limited the possibility of conducting a more structured or quantitative comparative synthesis. This variability also constrains the comparability of findings across studies and limits the ability to draw conclusions regarding the relative effectiveness of different assessment strategies.

Additionally, the predominance of studies conducted in high-income countries may affect the transferability of findings to low- and middle-income settings, where educational structures, resources and population health needs may differ substantially.

Finally, the conceptual classification proposed in Tables 3 and 4, although grounded in the data, involves a degree of analytical interpretation that may be subject to biases inherent to the narrative synthesis process.

## Conclusions

The available evidence indicates that assessment of public health competencies in undergraduate medical education is in a process of evolution towards more integrated, contextualised models aligned with contemporary competency-based education approaches. The findings show a progressive transition from strategies centred on knowledge measurement towards performance-based approaches in real contexts, particularly in community and clinical scenarios. Likewise, the growing incorporation of social determinants of health as a structuring axis of assessment reflects an expansion of the training paradigm towards a more comprehensive understanding of health, in which social, cultural and structural factors acquire a central role.

Nevertheless, this evolution is accompanied by marked methodological heterogeneity and absence of standardised evaluative frameworks, which limits comparability between studies and consolidation of the field. In this context, it is essential to advance towards development of programmatic, longitudinal and multimodal assessment strategies, as well as towards validation of instruments that allow consistent measurement of public health competencies. Equally, it is necessary to promote evidence generation in underrepresented contexts, in order to ensure global applicability of findings and strengthen training of physicians capable of responding to contemporary challenges of health systems. As an initial step towards standardisation, medical schools should work collaboratively to develop consensus-based competency frameworks and core assessment principles adaptable to diverse educational contexts.

## Data Availability

All data generated or analysed during this study are included in this published article and its supplementary materials. The data extraction matrix used in this review is available from the corresponding author upon reasonable request.

## Abbreviations

CBME: Competency-Based Medical Education
OSCE: Objective Structured Clinical Examination
OSF: Open Science Framework
PRISMA-ScR: Preferred Reporting Items for Systematic Reviews and Meta-Analyses Extension for Scoping Reviews
SDOH: Social Determinants of Health
SSDH CAT: Social and Structural Determinants of Health Curriculum Assessment Tool
